# Genetic and functional enrichments associated with *Enterococcus faecalis* isolated from the urinary tract

**DOI:** 10.1128/mbio.02515-23

**Published:** 2023-11-14

**Authors:** Belle M. Sharon, Amanda P. Arute, Amber Nguyen, Suman Tiwari, Sri Snehita Reddy Bonthu, Neha V. Hulyalkar, Michael L. Neugent, Dennise Palacios Araya, Nicholas A. Dillon, Philippe E. Zimmern, Kelli L. Palmer, Nicole J. De Nisco

**Affiliations:** 1 Department of Biological Sciences, University of Texas at Dallas, Richardson, Texas, USA; 2 Department of Urology, University of Texas Southwestern Medical Center, Dallas, Texas, USA; University of Pittsburgh School of Medicine, Pittsburgh, Pennsylvania, USA

**Keywords:** urinary tract infection, host-microbe interactions, adaptation, genomics, hybrid assembly, *Enterococcus*

## Abstract

*Enterococcus faecalis* is the leading Gram-positive bacterial species implicated in urinary tract infection (UTI). An opportunistic pathogen, *E. faecalis* is a commensal of the human gastrointestinal tract (GIT), and its presence in the GIT is a predisposing factor for UTI. How *E. faecalis* colonizes and survives in the urinary tract (UT) is poorly understood, especially in uncomplicated or recurrent UTI. The UT is distinct from the GIT and is characterized by a sparse nutrient landscape and unique environmental stressors. In this study, we isolated and sequenced a collection of 37 clinical *E. faecalis* strains from the urine of primarily postmenopausal women. We generated 33 closed genome assemblies and 4 highly contiguous draft assemblies and conducted a comparative genomics analysis study to identify genetic features enriched in urinary *E. faecalis* with respect to *E. faecalis* isolated from the human GIT and blood. Phylogenetic analysis revealed high diversity among urinary strains and a closer relatedness between urine and gut isolates than blood isolates. Plasmid replicon (rep) typing further underscored possible UT-GIT interconnection, identifying nine shared rep types between urine and gut *E. faecalis*. Both genotypic and phenotypic analyses of antimicrobial resistance among urinary *E. faecalis* revealed infrequent resistance to the front-line UTI antibiotics nitrofurantoin and fluoroquinolones and no vancomycin resistance. Finally, comparing gene presence and absence among urinary and gut strains, we identified 19 candidate genes enriched among urinary strains. These genes are involved in the core processes of sugar transport, cobalamin import, glucose metabolism, and post-transcriptional regulation of gene expression.

**IMPORTANCE:**

Urinary tract infection (UTI) is a global health issue that imposes a substantial burden on healthcare systems. Women are disproportionately affected by UTI, with >60% of women experiencing at least one UTI in their lifetime. UTIs can recur, particularly in postmenopausal women, leading to diminished quality of life and potentially life-threatening complications. Understanding how pathogens colonize and survive in the urinary tract is necessary to identify new therapeutic targets that are urgently needed due to rising rates of antimicrobial resistance. How *Enterococcus faecalis*, a bacterium commonly associated with UTI, adapts to the urinary tract remains understudied. Here, we generated a collection of high-quality closed genome assemblies of clinical urinary *E. faecalis* isolated from the urine of postmenopausal women that we used alongside detailed clinical metadata to perform a robust comparative genomic investigation of genetic factors that may be involved in *E. faecalis* survival in the urinary tract.

## INTRODUCTION

*Enterococcus faecalis*, a Gram-positive bacterium, is an increasingly frequent cause of urinary tract infection (UTI), especially in complicated or recurrent cases (1–5). While a strong body of work has elucidated virulence mechanisms associated with complicated enterococcal UTI, namely, catheter-associated UTI, little is known about how enterococci cause uncomplicated and recurrent UTI (6–11). *E. faecalis* natively inhabits the human gastrointestinal tract (GIT) but is an opportunistic pathogen (12, 13). *E. faecalis* GIT colonization is proposed to be a predisposing factor to UTI since periurethral contamination by gut bacteria is an important route of infection (14–16). This idea is supported by a reported association between gut enterococci abundance and *Enterococcus* UTI (14). In addition, premenopausal recurrent UTI (rUTI) patients were found to have a greater frequency of infection with endogenous gut microbes, specifically *Escherichia coli* and *E. faecalis* (17–19). Despite the association of *E. faecalis* with rUTI, little is known about the mechanisms by which *E. faecalis* colonizes the urinary tract in the absence of a catheter and the genetic determinants that enable its persistence.

Enterococci demonstrate great adaptability to thrive in stressful environments (13, 20). Urine, as compared to the gut environment, is a nutrient-limited medium characterized by high osmolarity, limited nitrogen and carbohydrate availability, moderate oxygenation, and low pH (21–23). Urine is also antimicrobial, composed of high concentrations of urea and antimicrobial proteins (23). Survival in the urinary tract despite environmental pressures and antibiotic intervention suggests that *E. faecalis* urine isolates may be specialized genetically or phenotypically. However, understanding the genetic factors necessary for *E. faecalis* growth in the urinary environment is limited.

To date, studies of *E. faecalis* urinary fitness are limited and focus on OG1RF (oral isolate) or V583 (blood isolate) (24). Previous studies of *E. faecalis* urinary fitness were conducted using well-studied *E. faecalis* strains OG1RF, V583, and MMH594 grown in pooled urine (25–28). These identified differential expression of genes encoding a sucrose phosphotransferase system (PTS), the *lutABC* operon for L-lactate metabolism, an amino acid ABC transporter, and *efaCBA* for manganese scavenging during growth in urine (28). However, no comprehensive study of a collection of clinical urinary *E. faecalis* isolates has been published. Although various virulence factors have been proposed as pathogenicity markers, a virulence genotype to distinguish uropathogenic *E. faecalis* from other *E. faecalis* strains has yet to be proposed (12).

To gain a deeper understanding of the genetics of urinary *E. faecalis* strains, we generated a collection of high-quality genomic sequences of clinical urinary *E. faecalis* isolates and compared them to isolates from the human gut and blood—distinct anatomical sites that pose unique evolutionary pressures on *E. faecalis*. Our findings show that urinary strains are diverse, possessing a wide range of plasmid replicon types and exhibiting low antimicrobial resistance rates, as determined both by genotypic predictions and phenotypic assays. We find that vancomycin resistance, which is commonly studied in this genus, was absent from the urine group, and intermediate fluoroquinolone resistance was common but did not correlate with any known chromosomal mutations or resistance genes. Finally, we identified genes involved in carbohydrate transport and metabolism, as well as vitamin B12 import and post-transcriptional regulation of gene expression, as enriched among urinary isolates. Together, this work provides a resource of high-quality and well-curated urinary *E. faecalis* genomes and identifies candidate pathways that may be important for *E. faecalis* urinary tract colonization.

## RESULTS

### Collection of urinary *E. faecalis* strains isolated from the urine of postmenopausal women

The role of *E. faecalis* in uncomplicated UTIs or in the microbiome of asymptomatic women is not well understood. Nevertheless, *E. faecalis* is commonly isolated from the female urogenital tract (29–31). Indeed, secondary analysis of our recently published metagenomic study of the urinary microbiome of postmenopausal women revealed that *E. faecalis* was present in 57.3% of samples (32). In patients with active rUTI at the time of urine collection, *E. faecalis* was detected in 46.2% of samples. In women without UTI but with a history of rUTI, *E. faecalis* was detected in 62.5% of urinary microbiomes (32). These observations highlight the need to understand how *E. faecalis* adapts to this unique environment.

**FIG 1:**
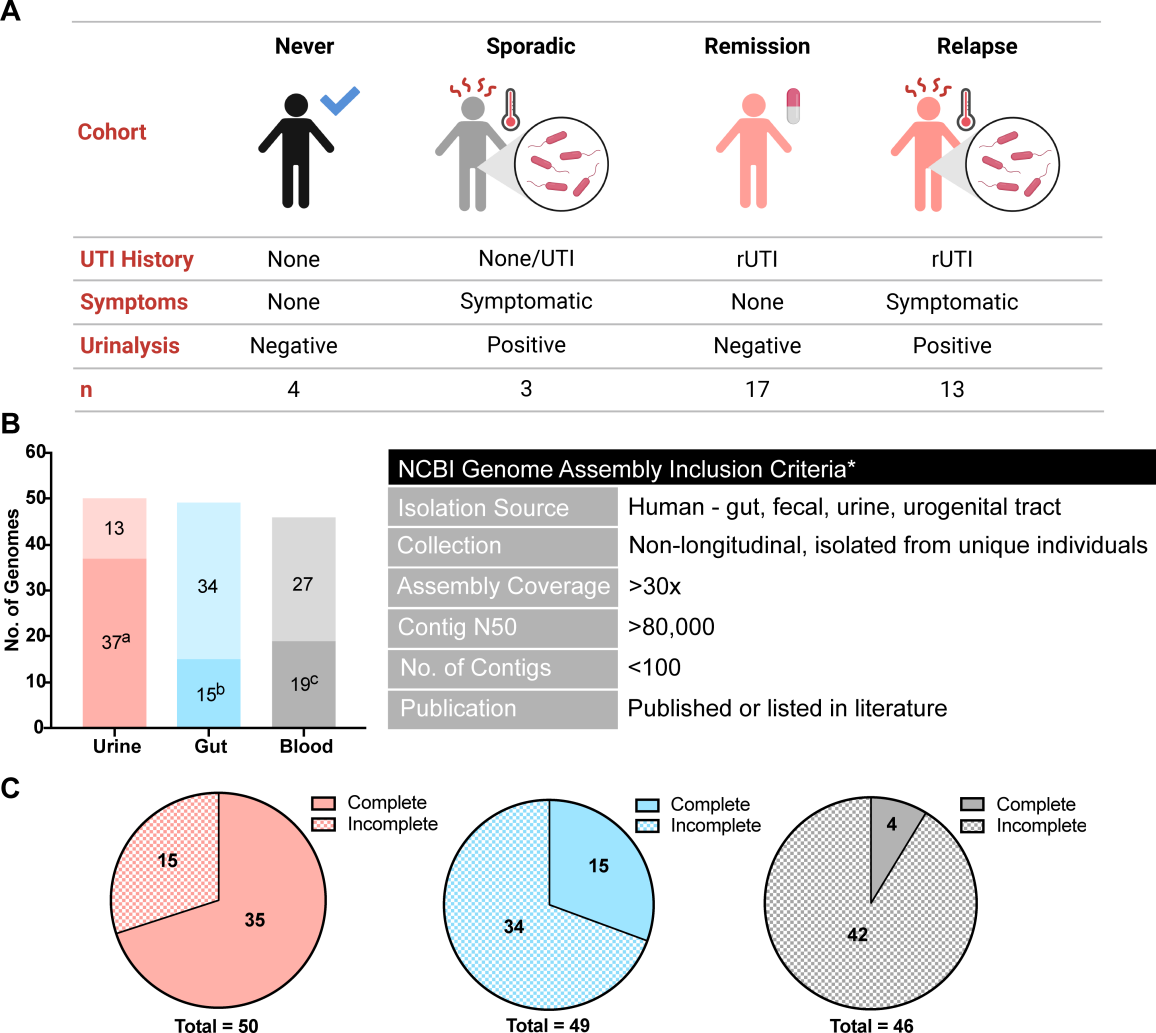
Clinical cohorts and an isolated collection of *Enterococcus faecalis*. (**A**) Patient cohorts were stratified based on patient UTI history, symptoms, and urinalysis results at the time of specimen collection. *n* denotes the number of isolates. (**B**) Number of isolate genomes from each isolation group. In the urine group, 13 isolates have been obtained from National Center for Biotechnology Information, while the remaining isolates have been sequenced as part of this study. ^a^Isolates sequenced as part of this study. ^b^Isolates from Palacios Araya et al.’s study (33, 34). ^c^Isolates from Van Tyne et al.’s study (24). Genome assembly criteria used for the selection of comparator genomes. *A couple of isolates reported in Table S3 do not meet one criterion but were included as they were reported in previous literature and met all other criteria. (**C**) Counts of complete and incomplete genome assemblies in the urine (pink), gut (blue), and blood (gray) isolation groups.

To begin to understand genetic features associated with urinary *E. faecalis* strains, we sequenced 37 unique *E. faecalis* strains isolated from the urine of postmenopausal women using both Illumina and Nanopore sequencing for the generation of closed or highly contiguous high-quality genome assemblies (Table S1). *E. faecalis* strains were isolated from the urine of consenting postmenopausal women who were recruited from a tertiary care center in Dallas, TX, USA, between October 2017 and April 2019 (Fig. 1A; Table S2). The 37 women were stratified into four cohorts based on their history of rUTI, UTI symptoms, and urine culture: never UTI (no clinical history of symptomatic UTI, *n* = 4), sporadic (no history of rUTI, current symptomatic UTI, *n* = 3), remission (history of rUTI, no current UTI, *n* = 17), and relapse (history of rUTI, current symptomatic UTI, *n* = 13). The median patient age was 72, with a median body mass index (BMI) of 27.5. Additional clinically relevant metadata were collected, including diabetes status, smoking, electrofulguration history, estrogen hormone therapy (EHT), non-steroidal anti-inflammatory drugs (NSAIDs), and antibiotics. In total, 8 women (21.6%) were diabetic, 19 had undergone electrofulguration of trigonitis (51.3%), 18 were prescribed EHT (48.6%), 11 were prescribed NSAIDs (29.7%), and 15 were prescribed antibiotics (40.5%), with 9 of those on current treatment courses. The most prescribed antibiotics were nitrofurantoin, followed by trimethoprim-sulfamethoxazole and fluoroquinolones (Table S2). Twenty-five specimens were polymicrobial (67.6%), seven were co-infections of *E*. coli and *E*. faecalis (18.9%), four were dominated by *E. faecalis* (10.8%), and one was an *E. faecalis* mono-infection (2.7%) (Table S2).

Among the 37 urinary genomes, 33 were closed, while 4 were highly contiguous with <21 contigs (Table S1). As of March 2023, a total of 131 complete genome assemblies of *E. faecalis* isolated from the human host were publicly available on the National Center for Biotechnology Information (NCBI). Of the 131 complete genomes, only 9 were clearly identified as associated with human urine. This collection, therefore, presents a focused group of high-quality urinary *E. faecalis* genomes that, coupled with clinical metadata, provides a valuable resource for the field of *E. faecalis* and *E. faecalis* UTI.

### Complete urinary genomes allow comparative analysis of urinary *E. faecalis* to gut and blood isolates

We hypothesized that specific genetic factors and phenotypes would be associated with urinary tract-colonizing *E. faecalis* strains when compared to *E. faecalis* strains colonizing different host niches. To test this hypothesis, we curated comparator genomes of *E. faecalis* strains isolated from the urinary tract (*n* = 50), gastrointestinal tract (*n* = 49), and blood (*n* = 46), which we term “isolation groups” (Fig. 1B; Table S3). Each isolation group is composed of publicly available genome assemblies obtained from NCBI that meet the inclusion criteria described in Materials and Methods (Fig. 1C). The majority of urinary strains (74%) were sequenced as part of this study (Fig. 1B); 31% of gut isolates were obtained from Dallas, TX, fecal surveillance (33, 34); and 41% of blood isolates were obtained from a Wisconsin hospital outbreak in the 1980s (24). Proportions of complete assemblies available in the gut (31%) and blood (9%) isolation groups are substantially lower than those in the urine group (70%) due to the genome availability at the time of curation (Fig. 1C).

### Phylogenetic and pangenome analyses reveal high diversity among urinary *E. faecalis* strains

**FIG 2:**
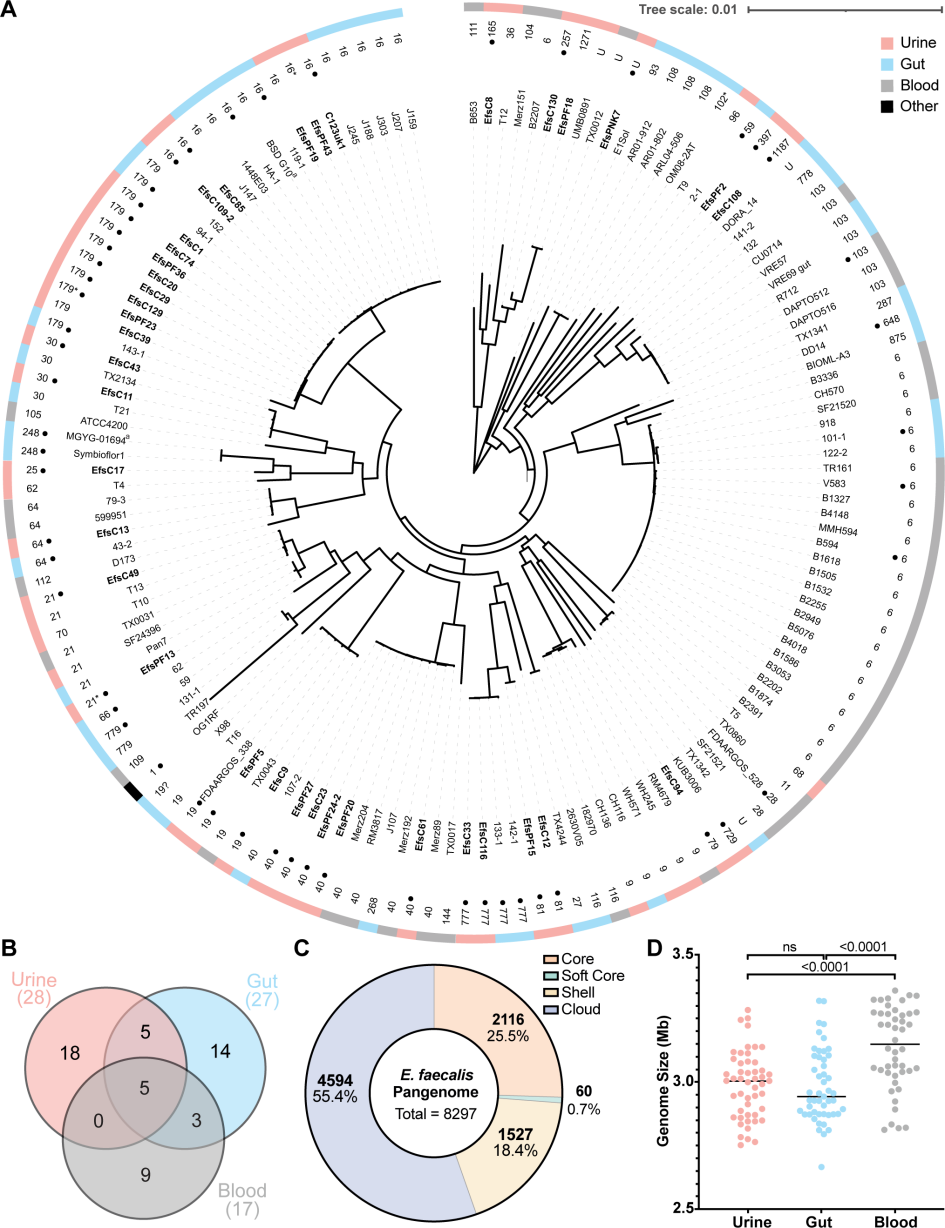
Phylogenetics, MLST, pangenome, and genome size distributions of urinary, gut, and blood *E. faecalis* isolates. (**A**) MinVAR-rooted maximum likelihood phylogenetic tree constructed from the core gene alignment of all strains in this study. Isolate names are listed on leaves. Dots represent the complete genome assembly. ST is depicted next to isolate names. U, unknown; *, ST has a novel allele; ?, ST uncertain. The ouutermost ring is color-coded by isolation source: urine (pink), gut (blue), blood (gray), and reference (black). (**B**) MLST Venn diagram depicting the total number of distinct sequence types in each of the isolation groups. Totals are listed below group names. (**C**) Pangenome analysis summary. Core genes are present in >99% of isolates; soft core genes are present in 95%–99% of isolates; shell genes are present in 15%–95% of isolates; and cloud genes are present in <15% of isolates. (**D**) Distributions of genome size in megabases of isolates per isolation group. Isolates are represented by dots, and the line represents the median. Statistical significance was determined using an ordinary one-way analysis of variance with multiple comparisons post-hoc. ^a^Isolate name has been shortened for simplicity. MGYG-01694, MGYG-HGUT-01694; BSD G10, BSD2780061688st3_G10.

Comparative analysis revealed that urinary strains are diverse (Fig. 2A). Urinary strains did not form unique clusters in a core gene phylogenetic tree. A large cluster of closely related ST6 blood isolates and a large mixed gut and urine cluster of ST179 and ST16 isolates were observed (Fig. 2A). Small urine-gut clusters as well as blood-gut clusters appear throughout the tree, suggesting closer relatedness between the urinary and gut strains than urinary and blood. The phylogeny largely follows the evolutionary estimates provided by multilocus sequence typing (MLST) (Fig. 2A). Urinary isolates are characterized by 28 different sequence types (STs) (Fig. 2B), with ST179 being the most common (14%). Among gut isolates, 27 different STs are present, with ST16 being the most common (20%). Interestingly, ST179 and ST16 are single locus variants of each other, differing by a synonymous variation in *xpt*. Blood isolates represent 17 STs and have a strong lineage bias for ST6, with 22 of the 46 isolates belonging to an ST6 hospital outbreak lineage (24). Despite this bias, this collection is representative of the genomic data available for bloodstream *E. faecalis*. We observed that urine isolates had the most unique STs (18) not found among isolates of the other groups, followed by gut (14) and blood isolates (9), and that urine and blood isolates do not share any STs that are absent from the gut group (Fig. 2B).

Pangenome analysis indicated that *E. faecalis* has an open pangenome consisting of a large proportion of accessory genes (soft core, shell, and cloud) and a small proportion of core genes (25.5%). In total, 8,297 genes make up the pangenome (Fig. 2C). Isolation source-specific pangenome analysis revealed a similar trend emphasizing the diversity among urinary isolates (Fig. S1). Analysis of genome size distribution showed that genomes from blood isolates are on average significantly larger than those from urine and gut (Fig. 2D). Conversely, no significant difference in average genome size was detected between urine and gut isolates. Furthermore, genome size was not significantly different between isolates of Dallas and non-Dallas origins (Fig. S1). These data indicate that urinary isolates are evolutionarily diverse, encompassing many STs and a wide range of genome sizes, and support the hypothesis that urine isolates originate from gut reservoirs (14, 15, 19, 35).

### Plasmid replicon typing suggests similarities in plasmid carriage between urinary and gut isolates

**FIG 3:**
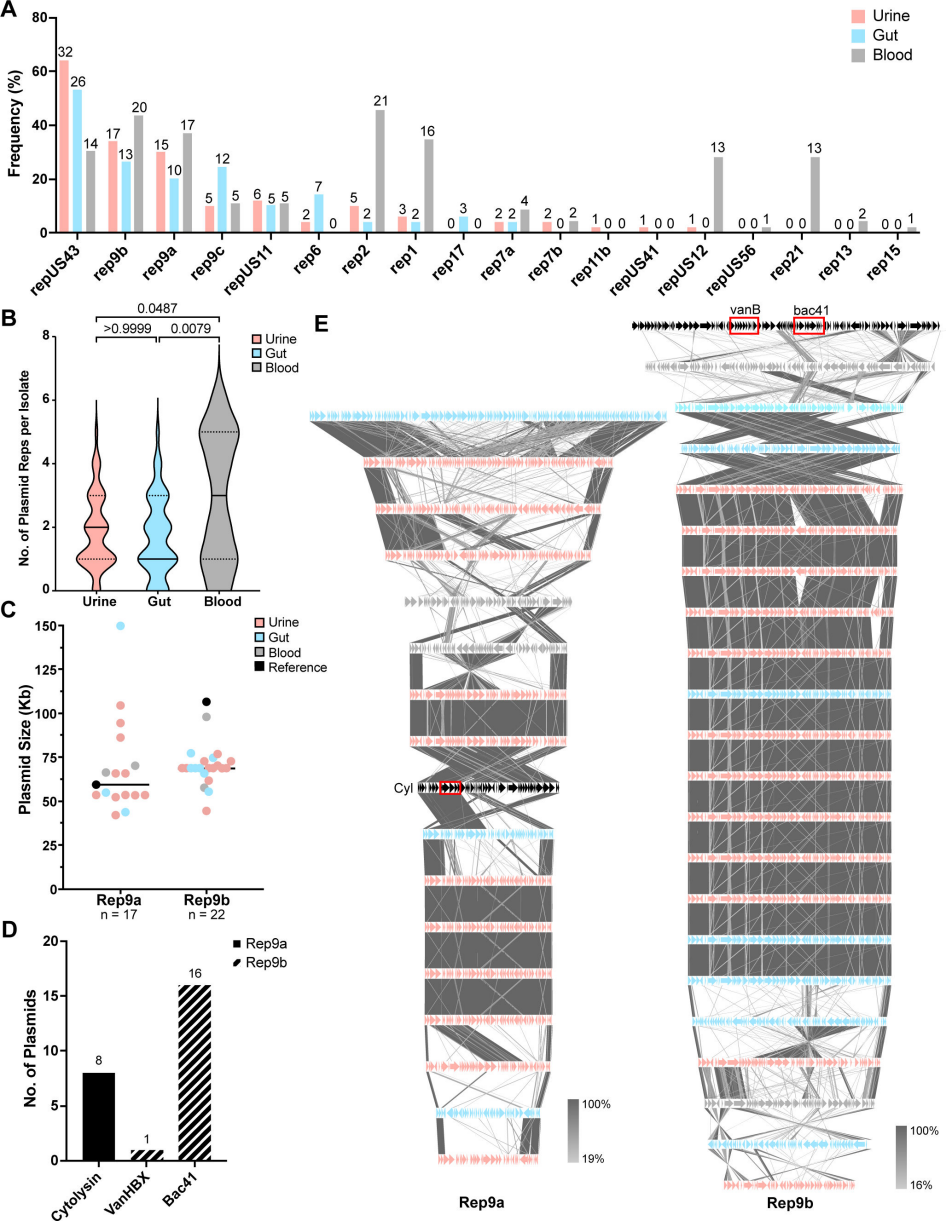
Rep type analysis and in-depth comparison of rep9a and rep9b. (**A**) Frequency (raw counts normalized to total group size) of all reps identified. Isolate counts are listed at the bar top. (**B**) Distribution of the number of plasmid reps per isolate in each isolation group. Statistical significance was determined using the Kruskal-Wallis test with multiple comparisons. (**C**) Size in kilobases of each rep9a and rep9b plasmid within complete genome assemblies. Each dot represents a single plasmid and is color-coded by isolation group. Rep9a *n* = 17, Rep9b *n* = 22. (**D**) Number of plasmids possessing cytolysin, vancomycin resistance operon VanHBX, and bacteriocin Bac41 as identified by sequence alignments. Bars are colored by the plasmid rep type associated with the locus. (**E**) tblastx alignments of rep9a (left, *n* = 17) and rep9b (right, *n* = 22) complete plasmids. Arrows denote coding sequences and are color-coded by plasmid isolation source: urine (pink), gut (blue), blood (gray), and reference (black). The reference rep9a plasmid is DS16 pAD1. The reference rep9b plasmid is pMG2200. Shaded lines between plasmid sequences are colored based on sequence identity (%). The loci of the cytolysin operon, the VanHBX operon, and bac41 are highlighted in the reference plasmid by red blocks.

Plasmids are drivers of evolution and virulence in bacteria (36). We hypothesized that urinary isolates possess characteristic conserved plasmid replicon (rep) types due to within-niche specialization. PlasmidFinder identified 18 unique rep types among all *E. faecalis* strains, 8 of which were present in all groups (repUS43, rep9b, rep9a, rep9c, repUS11, rep2, rep1, rep7a) (Fig. 3A; Table S4). A circular extrachromosomal element within EfsPF36 was not typeable by PlasmidFinder or NCBI PGAP. Prophage analysis determined that the extrachromosomal element, named here EfsPF36_phage01, is an intact prophage, phiFL2A (Table S4). The total number of replicons per isolate did not significantly differ between Dallas and non-Dallas isolates (Fig. S2).

Rep6 was the only rep present in urine and gut isolates, but not in blood. Rep7b and repUS12 were only present in urine and blood isolates but not in the gut. Of the rep types that were unique to a single isolation group, rep17 was only identified among gut isolates; rep11b and repUS42 were identified only in urine isolates; and repUS56, rep21, rep13, and rep15 were only identified in blood isolates (Fig. 3A). We found that the blood group possessed the total highest number of replicons (*n* = 134), followed by urine (*n* = 92) and gut (*n* = 82) (Fig. 3A). Additionally, the blood group had the highest median number of replicons per isolate (Fig. 3B).

The most common rep type was repUS43, found in 64% of urine, 53% of gut, and 30% of blood isolates (Fig. 3A). Analysis of the complete, closed genomes generated in this study revealed that repUS43 is chromosomally integrated and is proximal to a tetracycline resistance gene (*tetM*) (Table S4). The following two most prevalent replicons were rep9a and rep9b, which are associated with pheromone-responsive plasmids. rep9a is associated with pAD1 lineage plasmids known to encode the virulence factor cytolysin (37–39). Additionally, rep9b is associated with pMG2200, reported to encode vancomycin resistance (*vanHBX*) and a Bac41-type bacteriocin (40). We found that the identified rep9a plasmids were diverse in size, ranging from 149 kb to 42 kb, and only 47% encoded the cytolysin operon (Fig. 3C and D). Alignments of complete rep9a plasmids and the DS16 pAD1 reference, which was assembled as part of this study, further suggest that their genomic content varies widely (Fig. 3E; Fig. S3). Rep9b plasmids also have a broad size range from 106 to 44 kb; however, 40.9% of complete rep9b plasmids are approximately 68 kb (Fig. 3C). Alignments of complete rep9b plasmids and the pMG2200 reference indicate that *vanHBX* is only encoded in pMG2200, while 72.7% of rep9b plasmids encode the *bac41* operon (Fig. 3D and E).

### Pseudolysogenic extrachromosomal linear phages are enriched among geographically proximal isolates

**FIG 4:**
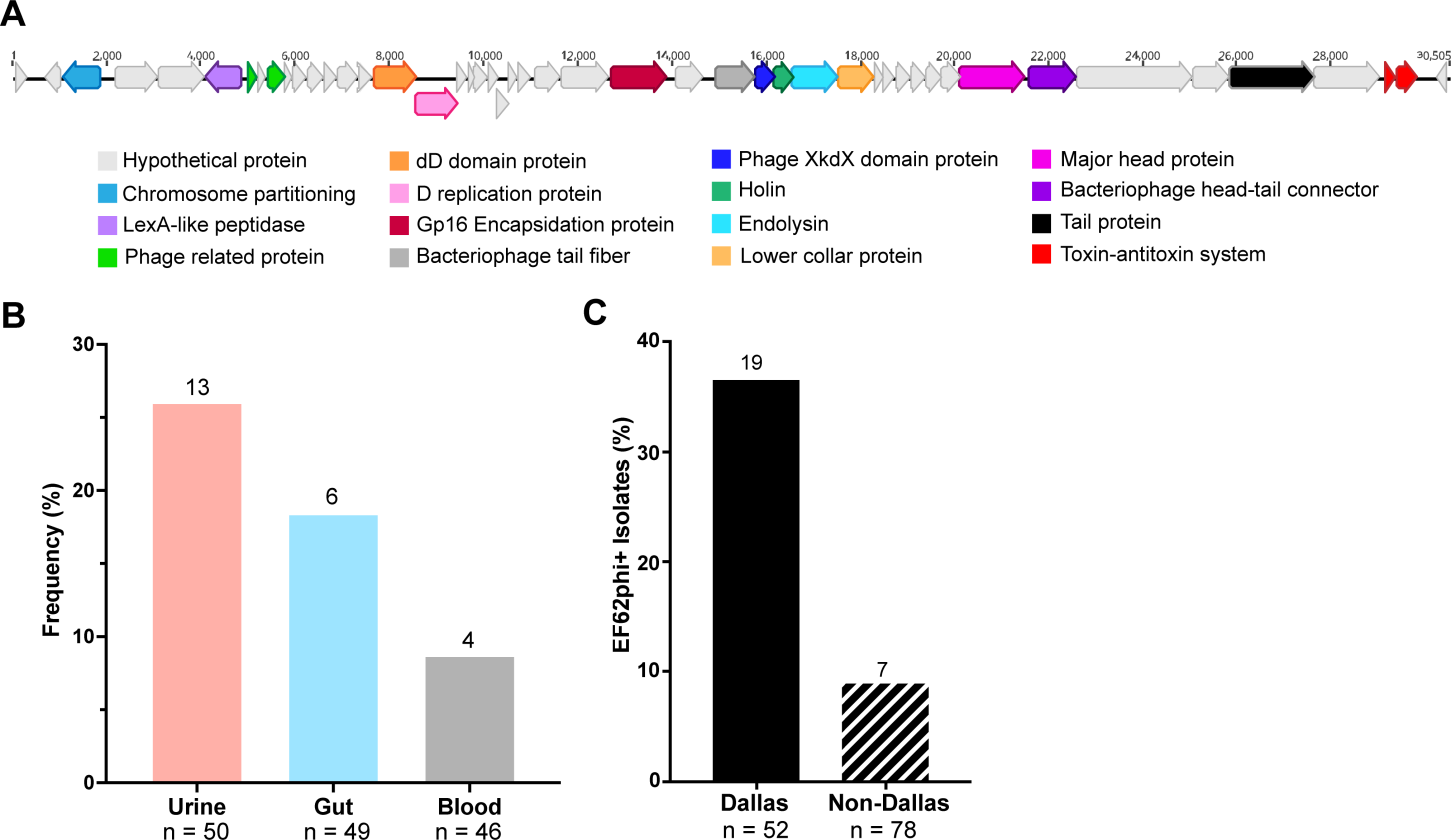
Extrachromosomal pseudolysogenic bacteriophage EF62phi prevalence in *E. faecalis*. (**A**) Complete genome of EF62phi with color-coded annotation of coding sequences. (**B**) Frequency of the presence of EF62phi within the genomes of each isolation group. Raw isolate counts are listed at bar tops. (**C**) Number of strains possessing EF62phi that were isolated from the Dallas (*n* = 52) versus non-Dallas (*n* = 78) areas. Raw isolate counts are listed at bar tops. Geographies were determined for a subset of genomes for which geographical metadata were available.

Complete genome assemblies of the urinary collection revealed a prevalent pseudolysogenic extrachromosomal linear phage (41). The phage, EF62phi, originally identified in *E. faecalis* 62 (SAMN02603509) isolated in Norway, encodes common structural phage components as well as a toxin-antitoxin system (Fig. 4A). Because EF62phi was identified in 13 (26%) of the urinary isolates but only in 18.4% of gut and 8.7% of blood isolates, we first hypothesized that EF62phi may be enriched in urine isolates (Fig. 4B; Table S4). However, EF62phi was commonly found in the gut strains also obtained from Dallas, TX. Thus, we hypothesized that EF62phi was instead associated with an isolate of geographical origin. Analysis utilizing available geographical metadata revealed that a greater percentage (36.5%) of *E. faecalis* isolated from the Dallas Metroplex in Texas, USA, harbored EF62phi than non-Dallas isolates (8.97%) (Fig. 4C).

### Antimicrobial resistance is not widespread among urinary *E. faecalis* isolates

**FIG 5:**
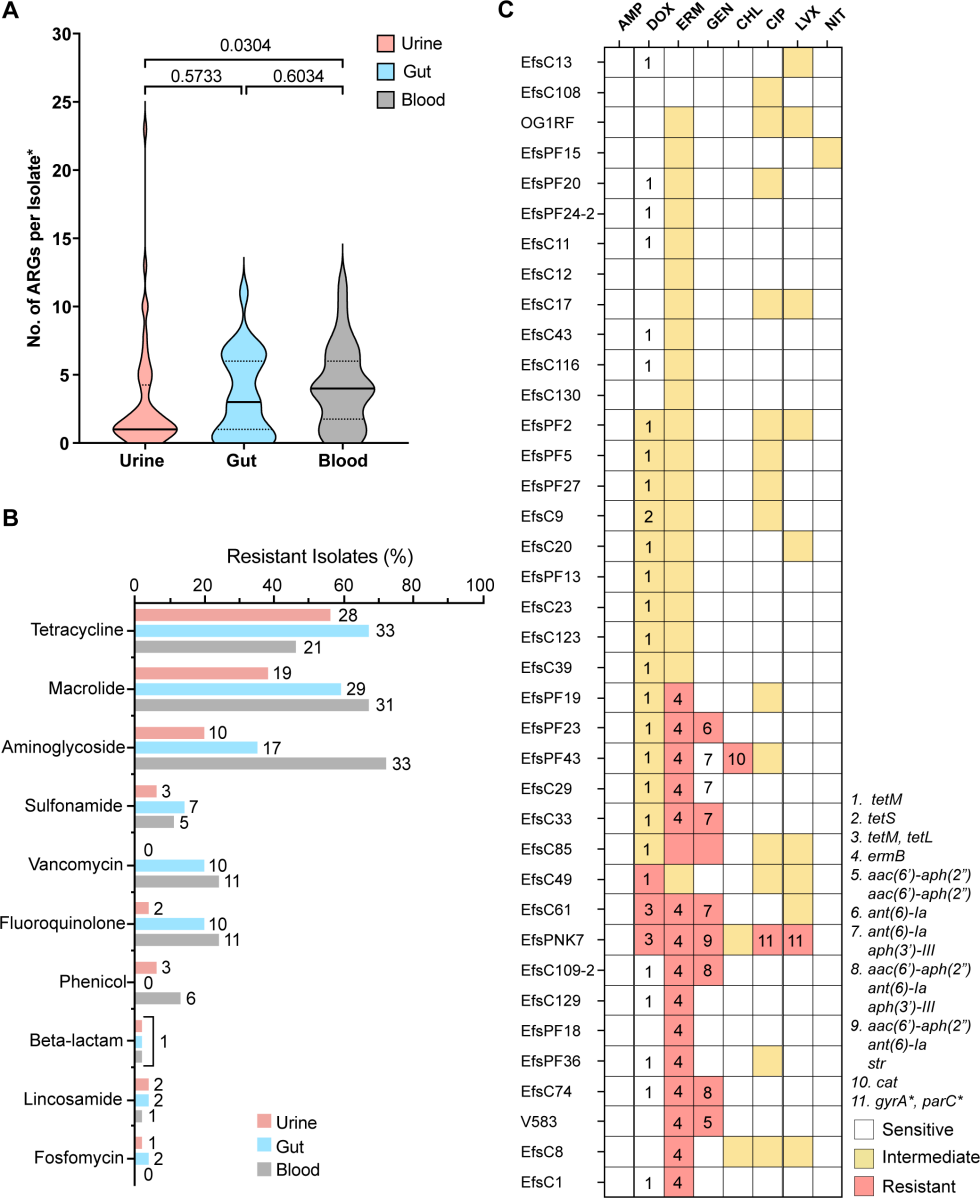
Antimicrobial resistance genes, chromosomal mutation predictions, and resistance phenotypes of urinary isolates. (**A**) Distribution of the total number of ARGs per strain in each isolation group. Each copy of a multi-copy ARG was counted. *Chromosomal mutations were counted as ARGs. Statistical significance was determined using the Kruskal-Wallis test with multiple comparisons. (**B**) Frequency of resistant isolates in each isolation source as predicted by ARG *in silico* analysis. Isolate counts are listed as bar ends. (**C**) Heatmap of resistance phenotypes assessed by disk diffusion and minimum inhibitory concentration assays. Numbers correspond to the presence of an ARG or mutation. Ubiquitous efflux pumps and intrinsic resistance genes are not depicted.

Antimicrobial resistance gene (ARG) analysis was used to predict *E. faecalis* resistance and assess differences between the isolation groups. We hypothesized that urinary isolates would be commonly resistant to UTI front-line therapies. Strikingly, the median number of ARGs, including known point mutations, per isolate was lowest in the urine group (Fig. 5A). To determine if this enrichment may be due to geographic location, we analyzed ARG counts between Dallas and non-Dallas isolates and found that the total number of ARGs did not significantly differ between Dallas and non-Dallas isolates (Fig. S2). Resistance was predicted for 11 drug classes and attributed to 31 unique ARGs or mutations in *E. faecalis* from all three isolation groups (Fig. 5B; Table S5). Several ubiquitous genes confer intrinsic resistance to antimicrobials in the species. These genes include *lsaABE* encoding multidrug resistance ABC-F subfamily efflux pumps, *dfrE* encoding a dihydrofolate reductase responsible for trimethoprim resistance, and genes encoding efflux pump components, *efrAB* and *emeA* (Table S5) (42–46). The most prevalent tetracycline resistance gene was *tetM*, which was found in 67% of gut, 56% of urine, and 46% of blood isolates. The presence of the *tetM* gene is directly correlated with the chromosomally integrated repUS43 locus (Spearman *r* = 0.7271, *P* < 0.0001) (Fig. 5B).

For aminoglycosides, fluoroquinolones, and vancomycin, urine isolates were predicted to be least frequently resistant, whereas blood isolates were predicted to be most frequently resistant. Aminoglycoside resistance was more common in blood isolates (72%) than in gut (35%) and urine (20%) (Fig. 5B). Fluoroquinolone resistance, as predicted by the presence of either an ARG or known *gyrA* and/or *parC* mutations, was similarly prevalent in gut (20%) and blood (24%) isolates but less frequent in urine isolates (4%) (Fig. 5B). Finally, vancomycin resistance was absent in the urine group but found in similar frequency in gut (20%) and blood (24%) isolates (Fig. 5B). However, the selection criteria for sequenced *E. faecalis* blood strains—mostly from hospital outbreaks and surveillance (24, 33)—may, in part, bias the number of vancomycin-resistant blood strains.

Although gene predictions offer insight, phenotypic assessments are imperative to confirm resistance. Phenotypic antibiotic resistance analysis of a large collection of urinary isolates has not been previously conducted; therefore, we analyzed the resistance phenotypes of 38 strains to 8 clinically relevant antibiotics from 7 drug classes (Fig. 5C). OG1RF and V583 were included as representatives of well-studied *E. faecalis* model strains. Resistant phenotypes among urinary isolates largely converged with gene predictions where specific resistance genes were present, whereas ubiquitous efflux pumps were not associated with resistance in all strains (Fig. 5C; Table S5).

Thirty resistant phenotypes were observed, of which 29 (96.6%) were attributed to an encoded ARG or known chromosomal mutations (Fig. 5C). Resistance to erythromycin was most widespread, with 42% of isolates demonstrating resistance, and was well-predicted by the *ermB* gene—15/16 resistant isolates encoded *ermB*. Gentamicin resistance was the second most prevalent with eight resistant isolates, and the presence of the *aac(6′)-aph(2″*) was associated with the most resistant phenotypes. Gentamicin resistance in EfsC85 was not explained by gene prediction, and the mechanism of resistance remains unclear (Fig. 5C).

The third most prevalent resistance was to doxycycline in the tetracycline class. The presence of *tetM* was not uniquely associated with resistance. In isolates encoding only *tetM*, isolate was resistant, 14 were intermediate, and 11 were susceptible. The co-occurrence of *tetM* and *tetL* was a more accurate predictor of doxycycline resistance (Fig. 5C). Chloramphenicol resistance was only predicted in one isolate and was associated with the *cat* gene. Finally, only one isolate was resistant to ciprofloxacin and levofloxacin due to the presence of known mutations in the quinolone resistance-determining regions (QRDRs) of the *gyrA* and *parC* genes (47) (Fig. 5C).

A total of 61 intermediate phenotypes were observed, of which 15 (39.4%) were attributed to the presence of a predicted ARG (Fig. 5C). The remaining 46 were detected for erythromycin (20), fluoroquinolones (ciprofloxacin, 14; levofloxacin, 9), and less commonly for chloramphenicol (2) and nitrofurantoin (1). Because of their clinical relevance, we further analyzed intermediate resistance to fluoroquinolones. We validated ciprofloxacin intermediate resistance by minimum inhibitory concentration (MIC) assay (Table S6) and confirmed that reported intermediate strains had ciprofloxacin MICs ≥2 and <4 μg/mL. Interestingly, no known point mutations or fluoroquinolone resistance ARGs were associated with the validated intermediate phenotypes (Fig. 5C).

### Genes involved in sugar transport, metabolism, and post-transcriptional stress responses are enriched in urinary *E. faecalis*

**FIG 6:**
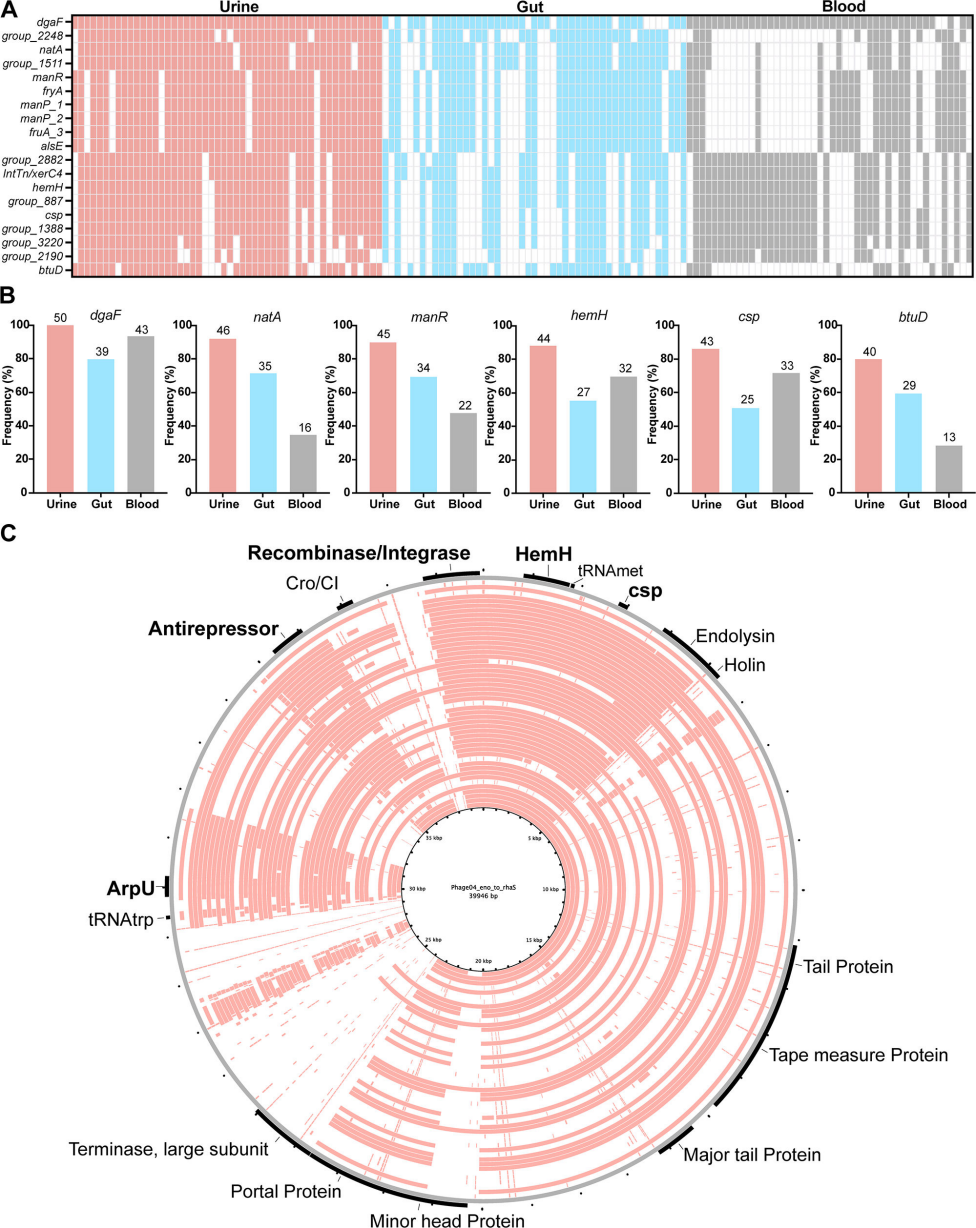
Candidate genes enriched among urinary *E. faecalis* isolates. (**A**) Presence/absence map of 19 candidate genes. Columns represent a single isolate, and colored blocks correspond to the isolation source: urine (pink), gut (blue), and blood (gray). Genes were identified by comparing urine and gut isolates; their presence in blood isolates is provided for reference. (**B**) Frequency of candidate genes within each isolation source. Isolate counts are listed at bar tops. (**C**) BLAST Ring Image Generator blastn alignment of urinary isolates (pink rings) phage04 integration region (*eno* to *rhaS*) to V583 phage04 reference (gray ring). Prophage annotations are listed, and enriched candidates are in bold. Isolate UMB0891 is not included in the alignment since the conserved region is not syntenic.

We next sought to identify gene enrichments within urinary versus gut strains of *E. faecalis*. Following inclusion cutoffs of >70% frequency in urine isolates and ³20% higher prevalence in urine than in the gut group, gene enrichment analysis identified 19 candidate genes as enriched in urinary isolates (Fig. 6A; Table S7). Among the 19 genes, 6 encode a PTS system operon predicted to be responsible for the transport of mannose and fructose (*manR*, *fryA*, *manP_1*, *manP_2*, *fruA*, *alsE*). Eight genes are located within a syntenic region of prophage phage 04 of V583 (locus EF1988–EF2043) (48, 49). These eight candidates include four hypothetical proteins (group_887, group_1388, group_2882, group_3220); two phage components, the ArpU family transcriptional regulator and a recombinase (*xerC*); and two intriguing, annotated candidates: *csp* encoding a cold-shock protein and *hemH* encoding a ferrochelatase (Fig. 6A and B). *Csp* is a unique member of the cold shock protein (CSP) family. There are seven *csp* orthologs in the *E. faecalis* pangenome, four of which are present in all *E. faecalis* strains. Among the *csp* genes that are not ubiquitous, the enriched *csp* candidate (EF1991) was found to be a unique cold shock family member present at higher frequency (86%) within urinary isolates (Fig. 6B; Figure S4A and B). The location of these genes within a prophage region suggests that they may have been acquired by horizontal gene transfer through a phage integration event. To determine if the enrichment of these eight prophage-associated genes was specific and not just a result of enrichment of the entire prophage, we aligned the phage region and performed blastn queries of candidate genes (Fig. 6C). These data, along with the presence/absence of data, suggest that the candidate genes are enriched independently of the intact phage because the phage structural genes, for example, are found at much lower frequencies among urine isolates than the eight enriched genes (Fig. 6C; Table S7).

The remaining five enrichment candidates include *dgaF*, *natA*, and *btuD* and two hypothetical proteins (group_1511, group_2248), one of which is directly downstream of *natA* (Fig. 6A and B). DgaF is an aldehyde lyase used in the Entner-Doudoroff (ED) pathway (50, 51). *NatA* is a sodium ABC transporter ATP-binding protein, while BtuD is a vitamin B12 import ATP-binding protein. In the *E. faecalis* pangenome, there are up to 19 distinct *btuD* orthologs, some of which are present in all isolates. A single *E. faecalis* may possess between 6 and 10 of the *btuD* orthologs, ranging in length from 768 to 2,340 bp. The enriched *btuD* candidate encodes a 297aa protein. Its length as well as sequence composition suggest that it is a unique family member (Fig. S4C). To test if the enrichment analysis may be confounded by isolation geography, we assessed differences in gene enrichment of representatives of the urinary strain-enriched genes between Dallas and non-Dallas isolates (Fig. S5). All candidates, with the exception of *btuD* and *natA*, showed no significant difference in enrichment between Dallas and non-Dallas isolates. Interestingly, both *btuD* and *natA* were significantly enriched (*P* = 0.0012 and *P* = 0.0084, respectively) among the Dallas isolates, suggesting that the observed urinary enrichment of these genes may indeed be confounded by isolation geography.

All hypothetical candidates were analyzed using the NCBI conserved domains database, and none were found to have conserved domains. However, blastp analysis found that candidate group_2248 was 100% identical to a T7SS effector LXG polymorphic toxin (accession numbers CP091889 and WP_238463980.1), and group_1511 downstream of *natA* was 99.1% identical to an annotated ABC transporter, permease protein (accession number HF558530). This, coupled with the respective lengths of *natA* and group_1511, suggests that the latter is *natB* (52). Candidate group_2882 of the phage04 region was 100% identical to an anti-repressor (accession number CP014949) (Table S7).

## MATERIALS AND METHODS

### Urinary *E. faecalis* strain isolation

Clean catch midstream urine was collected from female patients following informed consent and institutional review board approval (STU 082010-016, STU 032016-006, MR 17-120). Patients were stratified into four groups based on their UTI history, symptoms, and urine culture at the time of collection: never, sporadic, rUTI remission, and rUTI relapse. Here, rUTI is defined as ≥2 symptomatic UTIs within 6 months or ≥3 UTIs within 12 months (17, 53, 54). Patient age was >35, with the majority being postmenopausal (>55 years old). Additional clinical metadata, including BMI, smoking history, diabetes status, electrofulguration of trigonitis history, and prescription of EHT, NSAIDs, and antibiotics, were recorded (Table S2). Urinary *E. faecalis* isolates were cultivated from urine and preliminarily identified by plating 100 or 10 µL of urine on CHROMagar Orientation and incubating at 37°C overnight. Species identity was confirmed by PCR of the 16S rRNA gene and Sanger sequencing, followed by megablast queries at default thresholds as described previously (55–58).

### Urinary, gut, and blood *E. faecalis* strain genome collection

A-priori power analyses using one-way analysis of variance and Fisher’s exact test (G*Power) were conducted to determine the sample size for the comparative analysis of two or three groups (59). This analysis determined that to detect an effect size of 0.4 with a power of 0.8, a minimum of 41 genomes were needed in each isolation group. A total of 146 *E. faecalis* strains were included in this study: 50 urine isolates, 49 gut isolates, 46 blood isolates, and OG1RF, a commonly studied oral *E. faecalis* (Table S3). Among the 49 gut isolates, 15 genomes of *E. faecalis* gut isolates obtained from patients in Dallas, TX, USA area hospitals were included as they represent geographically relevant comparator strains and are high-quality closed genomes (BioProject PRJNA800580). *E. faecalis* genome assemblies available as of 28 July 2020 were downloaded from the NCBI assembly database following stringent curation. Inclusion criteria were as follows: human host, isolated from the gastrointestinal tract (gut, fecal), blood, or urine; isolated from distinct individuals (non-longitudinal); meet assembly quality parameters of average coverage >30×, contig N50 >80,000, and fewer than 100 contigs. Finally, isolates from published research were prioritized.

### Genomic DNA isolation and sequencing

Genomic DNA (gDNA) was extracted using the Qiagen Blood & Tissue Kit and assessed for quality using a 260/280 nm absorbance ratio, fluorometry, and agarose gel electrophoresis. High-quality gDNA libraries were prepared for short-read Illumina and long-read MinION sequencing as described previously (56, 60). See detailed methods in the supplemental material.

### Genome annotation and sequence type predictions

Genome annotation was performed using Prokka v1.14.6 and NCBI Prokaryotic Genome Annotation Pipeline v6.4 with default parameters (61, 62). Multilocus sequence typing was performed using MLST v2.0 on the Center for Genomic Epidemiology (CGE) server with the *E. faecalis* configuration and Assembled or Draft Genome/Contigs as input (63, 64).

### Plasmid replicon analysis

Plasmid replicon types were predicted using PlasmidFinder v2.1 at default parameters (64–66). Extrachromosomal elements not typeable by PlasmidFinder were assessed using NCBI Conserved Domain Search at default parameters, PHASTER, and blastn queries of the nr/nt database (67–69). PLSDB v2021_06_23_v2 was used with both Mash screen and Mash dist search strategies to identify similar plasmids (70, 71). Alignment of complete plasmid sequences was conducted using Easyfig v2.2.2 with tblastx at default parameters (72). Reference plasmids representing rep9a (pAD1 from strain DS16, SAMN00809239) and rep9b (pMG2200, AB374546.1) were included in the analysis for comparison. Further alignment and visualization were conducted using BLAST Ring Image Generator v0.95 with blastn at default thresholds.

### Pangenome and phylogenetic analyses

Pangenome analysis was conducted using Panaroo v1.2.10 with the merge-paralog option selected and core gene alignment conducted using mafft (73, 74). Reference pan-genome sequences are available in Data S1. Core gene alignment output was then used to construct a phylogenetic tree with IQ-TREE using the GTR + F + I + I + R6 model with ultrarapid bootstrapping (1,000 inferences) (75–77). A model of nucleotide substitution was selected with the IQ-TREE model (77). The phylogenetic tree was rooted using min-VAR rooting with FastRoot v1.5 and visualized with iTOL (78, 79).

### Gene enrichment analysis and functional annotation

Gene enrichment analysis was conducted using Scoary v1.6.16 (80). Genes with a *P*-value <0.05 were retained for further analysis (Table S7). Candidates were considered enriched if they were present in >70% of urine isolates and were at least 20% more prevalent in urine isolates than in the gut. Since the urine group is primarily composed of isolates sequenced in this study (74%), it was imperative to apply stringent cutoffs to ensure biological significance. Candidates were validated using blastn to confirm that paralogs were not erroneously split and account for highly conserved homologs. Hypothetical candidate genes were further assessed using NCBI Conserved Domain Search, PSORTb v3.0.3, blastn, and blastp queries to elucidate potential function (68, 69, 81). Allele sequence alignments were conducted using nucleotide MUSCLE v3.8.425 alignment at default parameters in Geneious Prime v2022.1.1 (82, 83).

### Antimicrobial resistance gene prediction

Antimicrobial resistance genes and point mutations were predicted using ResFinder v4.0 on CGE with the *E. faecalis* database at default parameters (84, 85). ABRicate v1.0.1 was used to query the CARD database at 70% coverage and 70% identity thresholds to validate ResFinder findings and identify additional resistance determinants (86, 87).

### Antimicrobial resistance phenotype assessment

Resistance phenotypes were assessed by Kirby-Bauer disk diffusion, as described previously, on brain-heart infusion (BHI) agar and following the established breakpoints of the Clinical and Laboratory Standards Institute (CLSI) (88–90). All ciprofloxacin phenotypes and intermediate or resistant gentamicin and chloramphenicol phenotypes were further validated using the MIC microdilution assay in BHI broth per CLSI breakpoints using the HT-MIC workflow as previously described (91). Further details can be found in the supplemental material.

## DISCUSSION

In this study, we analyzed the genomes of urinary, gut, and blood *E. faecalis* isolates to identify genetic features and functions associated with *E. faecalis* strains colonizing the urinary tract. A collection of 37 clinical urinary isolates was obtained from predominately postmenopausal women, sequenced, and hybrid-assembled to generate closed or highly contiguous genome assemblies. This collection provides a tool for further research in the fields of *E. faecalis* genomics and *E. faecalis* UTI. Our analysis of these and additional curated, publicly available *E. faecalis* genomes provides comprehensive insight into niche-specific population genetics, mobile genetic elements, antimicrobial resistance, and gene enrichments.

Here, we sought to test the hypothesis that urinary *E. faecalis* possesses gene enrichments that may be associated with colonization of the urinary environment. We postulated that urinary isolates should be genetically conserved, encode characteristic plasmids, be resistant to similar antimicrobials, and encode genes that may aid in survival within the urinary tract. Additionally, as the gut-bladder axis becomes further defined in UTI research, we estimated that urinary isolates will resemble gut isolates more than blood isolates (15, 19, 35).

Our findings reveal that urinary *E. faecalis* is diverse, and no lineage emerges as strongly associated with the urinary niche. The most frequent ST in the urinary group, ST179, is a single-locus variant of the most common ST in the gut group, ST16. Strains belonging to these two STs form a large phylogenetic cluster and may support the gastrointestinal origin of urinary isolates. Blood isolates demonstrate an expected lineage bias for ST6 (24). As genome availability is often a reflection of study selection criteria, ST6 blood isolates have been primarily associated with hospital outbreaks and represent a large collection of publicly available strains (24). We recognize that this introduces bias to the analysis and cautiously draw conclusions from urinary versus blood comparisons.

Plasmid replicon analysis did not identify strong associations between rep type and isolation source. With the exception of two rep types (rep11b, repUS41), which are both present in a single isolate, EfsC94, no rep types were found to be strictly associated with urinary isolates. Most rep types were represented in all three isolation groups, with pheromone-responsive rep9a, rep9b, and rep9c being among the most prevalent. RepUS43 was the most common rep type, and our analysis found it to be chromosomally integrated within all complete genome assemblies assessed—an insight not previously reported in the literature. Furthermore, repUS43 was positively correlated with the presence of the tetracycline resistance gene *tetM*, suggesting hat this resistance gene may be widespread due to a recombination event with a repUS43 plasmid in a common ancestor. Rep typing offered an insight into the plasmid content of urinary *E. faecalis* but not without limitations. Alignments of complete rep9a and rep9b plasmids revealed that plasmids of these rep types can vary widely in terms of size as genes encoded. Rep typing, thus, may be useful for certain conserved replicons but should be utilized with caution when considering plasmid function.

Antimicrobial resistance *in silico* predictions indicated that urinary *E. faecalis* encodes fewer ARGs than its gut or blood counterparts. In all but two drug classes, urinary isolates encoded ARGs less frequently than gut and blood isolates. Acquired ARGs or mutations predicted to confer fluoroquinolone or nitrofurantoin resistance were not prevalent in urine isolates. Phenotypic *in vitro* assessments of the antibiotic susceptibility of urinary isolates demonstrated a similar trend in which resistance was not widespread and intermediate phenotypes were more prevalent. Intermediate fluoroquinolone resistance was relatively common, but the mechanism remains unclear. Uncharacterized *gyrA* and *parC* mutations were present in some strains, although not within the QRDR regions. A limitation of this study is that the strains analyzed were isolated from the same clinic and are likely not fully representative of different locations, clinical practices, and demographic groups.

To address the hypothesis that urinary *E. faecalis* possesses gene enrichments that may be associated with urinary tract colonization, we conducted gene enrichment analysis comparing urinary and gut isolates. Applying stringent inclusion cutoffs, we identified 19 candidate genes enriched in the urinary group. We then further narrowed down candidates to those that did not demonstrate enrichment in Dallas versus non-Dallas isolates to control for a potential confounder of isolate geography. Of note, we found an enrichment of genes encoding a mannose/fructose PTS system. This may be relevant to the urinary niche because the glucose concentration in urine is often low (0.2–0.6 mM) (92), and therefore this operon may help *E. faecalis* utilize alternate carbon sources that are more abundant in urine, like mannose (28). Intriguingly, D-mannose supplements are routinely advised for UTI prophylaxis, as studies suggest that they may block colonization by uropathogenic *E. coli*, which adhere to the host urothelium via mannosylated cell surface proteins (93, 94).

Additional candidates like *dgaF*, which functions as a 2-keto-3-deoxygluconate 6-phosphate aldolase to form glyceraldehyde-3-phosphate and pyruvate similar to the Eda enzyme in the ED pathway and is found in all urinary isolates, may be important in *E. faecalis* growth under the environmental pressures of the urinary niche (95). The ED pathway produces less ATP than glycolysis but offsets this metabolic cost by requiring fewer enzymes. While the ED pathway is often used by aerobic Gram-negative bacteria, *E. faecalis* is one of the few facultative anaerobic Gram-positive species that possess it (50, 96). We postulate that *E. faecalis* may utilize the ED pathway during urinary colonization to conserve protein and therefore energy costs (97).

Finally, two enrichment candidates, *hemH* and *csp*, were localized within a prophage region. HemH is a ferrochelatase known to incorporate ferrous iron to form coproheme in Gram-positive bacteria (98). However, *E. faecalis* lacks the machinery to synthesize heme, and thus the role of this ferrochelatase remains unclear (99). Of particular interest is *csp*, encoding a CSP. CSPs are a common adaptive mechanism for stress and changes in environmental conditions. The first CSP, CspA, was identified in *E. coli* and later found to have nine homologs (CspA–CspI). These proteins, commonly expressed in response to temperature decreases, function as chaperones that prevent the formation of secondary structures in RNA transcripts, thereby allowing proper translation (100, 101). However, some CSPs are known to be non-cold-inducible. Four of the *E. coli* CspA family members are not induced by cold shock. Such CSPs have been reported to be involved in osmotic, oxidative, starvation, pH, and ethanol stress tolerance as well as host cell invasion (100, 101). An example of such adaptive CSP has been described by Michaux et al. (101), who suggested that the CspR of *E. faecalis* plays a role in virulence and persistence (101). The enriched *csp* gene highlighted by our analysis was shown previously to be upregulated in V583 grown in blood (27). However, the function of the CSP allele enriched in urinary isolates and its role in urinary fitness have yet to be characterized in *E. faecalis*.

## Data Availability

All raw sequencing data and assembled genomes produced as part of this work are available on the NCBI SRA and GenBank databases and can be accessed through BioProject PRJNA944190.

## SUPPLEMENTAL MATERIAL

Data S1 mbio.02515-23-s0001.csv Pangenome fasta file in csv format.

Supplemental Material mbio.02515-23-s0002.pdf Supplemental methods, figures, and references.

Legend mbio.02515-23-s0003.docx Legend for Data S1.

Table S1 mbio.02515-23-s0004.xlsx Urinary *E. faecalis* genome assembly metadata.

Table S2 mbio.02515-23-s0005.xlsx Clinical metadata.

Table S3 mbio.02515-23-s0006.xlsx All genome metadata.

Table S4 mbio.02515-23-s0007.xlsx Rep analysis metadata.

Table S5 mbio.02515-23-s0008.xlsx ARG and point mutation analysis.

Table S6 mbio.02515-23-s0009.xlsx MIC data.

Table S7 mbio.02515-23-s0010.xlsx Gene enrichment data.

ASM does not own the copyrights to Supplemental Material that may be linked to, or accessed through, an article. The authors have granted ASM a non-exclusive, world-wide license to publish the Supplemental Material files. Please contact the corresponding author directly for reuse.
