# Supplementary material for: Genetic and functional enrichments associated with Enterococcus faecalis isolated from the urinary tract
Source: mBio. 2023 Nov 14;14(6):e02515-23. doi: 10.1128/mbio.02515-23 (PMC10746210; doi:10.1128/mbio.02515-23)
Supplement: Supplemental Material — Supplemental methods, figures, and references. [file mbio.02515-23-s0002.pdf]

SUPPLEMENTAL MATERIAL

Genetic and functional enrichments associated with *Enterococcus faecalis* isolated from the urinary tract

Belle M. Sharon[a], Amanda P. Arute[a], Amber Nguyen[a], Suman Tiwari[a], Sri Snehita Reddy Bonthu[a], Neha V. Hulyalkar, Michael L. Neugent, Dennise Palacios Araya[a], Nicholas A. Dillon[a], Philippe E. Zimmern[b], Kelli L. Palmer[a], Nicole J. De Nisco[a,b]#.

[a] Department of Biological Sciences, University of Texas at Dallas, Richardson, Texas, USA

[b] Department of Urology, University of Texas Southwestern Medical Center, Dallas, Texas, USA

LIST OF CONTENTS

Supplemental Methods

Figure S1 - Isolation group pangenomes & genome size versus geography comparison.

Figure S2 - Replicon and ARG counts versus geography comparisons

Figure S3 - DS16 pAD1 plasmid sequence fragments alignment

Figure S4 - *Csp* and *BtuD* paralog alignments

Figure S5 - Geographical location association with representative enrichment candidates

Supplemental References

SUPPLEMENTAL METHODS

Genomic DNA Isolation and Sequencing

High quality gDNA libraries were prepared for short-read sequencing using the Nextera DNA Flex library prep kit and sequenced using 2x150bp paired-end sequencing on Illumina NextSeq 500. Long-read sequencing libraries were prepared using Oxford Nanopore Technologies (ONT) ligation sequencing kit (SQK-LSK109) and barcode expansion kits EXP-NBD104 and EXP-NBD114 and sequenced using R9 FLO-MIN106 flow cells on ONT MinION as described previously (1, 2). Sequencing reads were subject to quality filtering and trimming using CLC Genomics Workbench v12.0.3 and NanoFilt v2.6.0 to discard short reads with Phred score <20 and length <15bp, and long reads with Phred score <7 and length <200bp, respectively (3). Genomes were assembled using Unicycler v0.4.8 at default parameters (4-7). Incomplete assemblies were later reassembled using Unicycler v0.5.0 at default parameters or bold mode to obtain the most complete assembly (fewest contigs). Genome completeness was assessed using CheckM v1.0.18 lineage workflow with the Lactobacillales order marker gene set on KBase v1.4.0 (8, 9). Draft genomes contig N50 and quality was assessed using QUAST v5.0.2 (10). All genomes have been deposited to NCBI via BioProject PRJNA944190. Accession numbers are provided in **Supplemental Table S2**.

DS16 pAD1 de novo Assembly

A complete pAD1 plasmid from DS16 was assembled using raw sequence read data of DS16 (SRX158205) using PlasmidSPADES v3.15.2 (11). The complete pAD1 plasmid assembly was validated using global nucleotide alignment with free end-gaps in Geneious Prime v2022.1.1 using publicly available and previously published pAD1 fragments (L01794.1, X62658.1, X62657.1, L19532.1, AB007844.1, X96977.1, X17214.1, AF343839.1, U00681.1) (12-19).

Antimicrobial Resistance Phenotype Assessment

Resistance phenotypes were assessed by Kirby-Bauer disk diffusion on Brain-Heart Infusion (BHI) agar to 8 representative antibiotics including: Ampicillin (AMP), Doxycycline (DOX), Erythromycin (ERM), Gentamicin (GEN), Chloramphenicol (CHL), Ciprofloxacin (CIP), Levofloxacin (LVX), and Nitrofurantoin (NIT). Briefly, Antibiotic disks were prepared by aliquoting 10 uL of antibiotic stock (GEN 1 mg/ml, AMP 1 mg/ml, CIP 0.5 mg/ml, LVX 0.5 mg/ml, ERM 1.5 mg/ml, CHL 3 mg/ml, NIT 30 mg/ml, DOX 3 mg/ml) onto the disk. Vehicle control disks were prepared similarly. Strains were streaked from glycerol stocks onto CHROMagar and incubated overnight at 37°C. Single isolated colonies were inoculated into 3 mL Brain-Heart-Infusion broth and incubated for 16 – 18 hours, then normalized to 0.5 McFarland standard, washed and resuspended in sterile 1X Phosphate-Buffered Saline (PBS). 150 µL of standardized culture were pipetted onto 150_mm BHI Agar plates and spread using sterile glass beads. Plates were dried before disks were placed on the agar. *Escherichia coli* ATCC25922 was used for quality and vehicle controls. Plates were incubated inverted overnight and antimicrobial susceptibility was evaluated by measurement of the zone of inhibition per the established zone diameter breakpoints of Clinical and Laboratory Standards Institute (CLSI) (20-22).

All Ciprofloxacin phenotypes and intermediate or resistant Gentamicin and Chloramphenicol phenotypes were further validated using Minimum Inhibitory Concentration (MIC) microdilution assay in BHI broth per CLSI breakpoints. MIC was measured using the HT-MIC workflow to determine the minimum inhibitory concentration of antibiotic required to inhibit 90% of the growth of untreated controls (MIC90) as previously described (23). In brief, 96-well 10x antibiotic master plates were prepared from antibiotic stocks using the Opentrons OT2 robot, and 30 µL was transferred to each well from the master plate to the test plates. Strains were first grown shaking at 200 rpm overnight at 37°C in a Innova S44 orbital shaker. The next day, 50 µL of each overnight culture was inoculated into fresh BHI and grown shaking at 200 rpm at 37°C until mid-log phase was reached, as determined by an OD600nm of approximately 0.4 or ~10^8 CFU/mL. Mid-log

phase cultures were diluted to an OD600 of 0.002 (~10^5 CFU/mL) and 270 μL of each culture was added to a separate well of the 96-well test plate already containing 30 μL of dispensed antibiotic. The test plates were covered with lids to prevent evaporation and were incubated for approximately 20 hours shaking at 200 rpm at 37°C. After incubation, the test plates were removed, and the OD600 of each well was measured on a Biotek Synergy H1 plate reader, using BHI as a blank. Measured OD600 values were used for MIC90 determination.

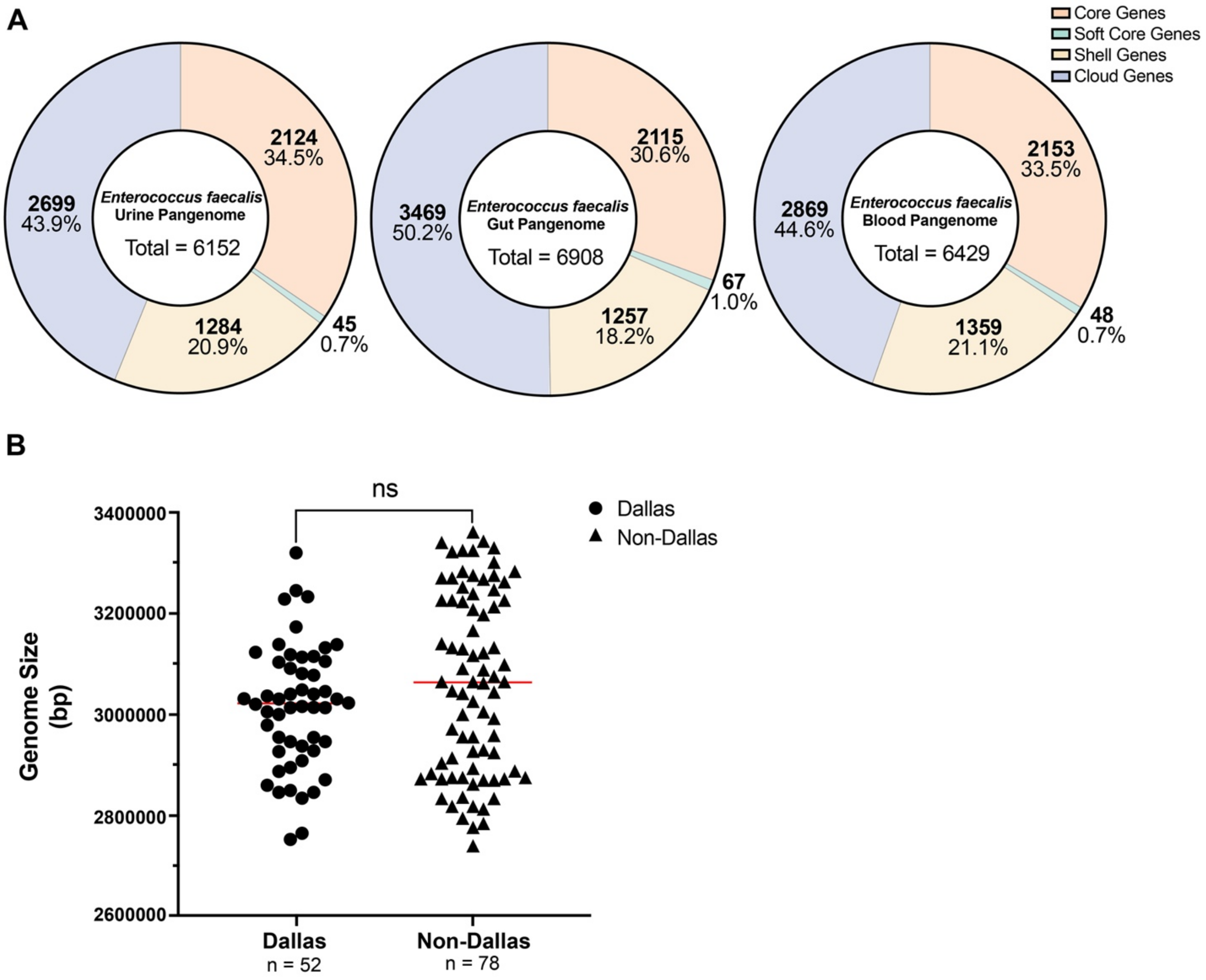

Figure S1. Isolation group pangenomes & genome size versus geography comparison. (A) Pangenome analysis of isolates by isolation group. Core genes present in >99% of isolates, Soft Core genes present in 95-99% of isolates, Shell genes present in 15-95% of isolates, and Cloud genes present in <15% of isolates. **(B)** Mann-Whitney statistical comparison of genome size and isolate geography. ns – not significant.

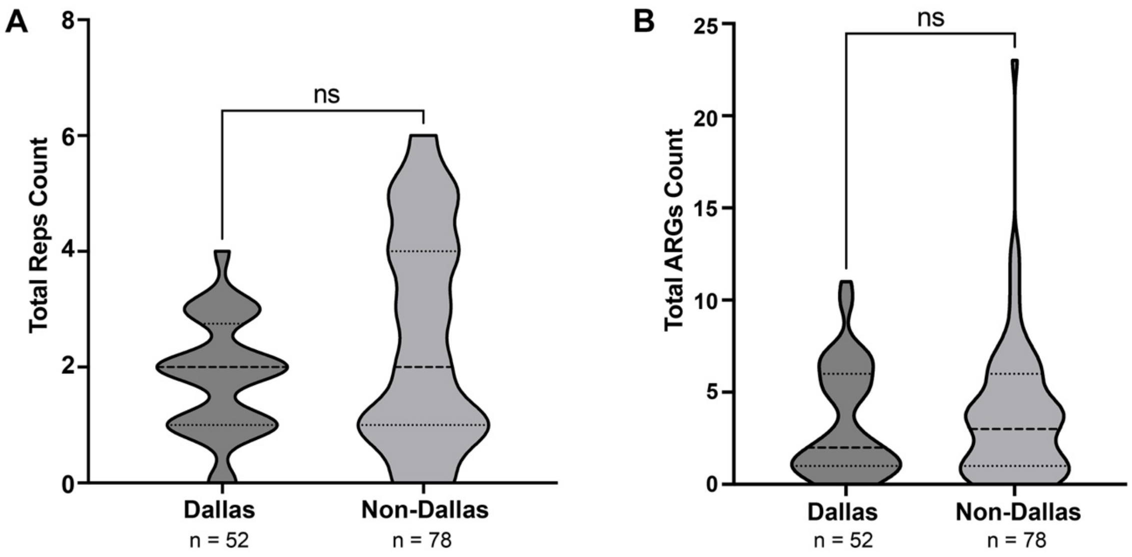

Figure S2. Replicon and ARG counts versus geography comparisons. Mann-Whitney statistical comparison for total rep count **(A)** and total ARGs, excluding ubiquitous ARGs **(B)** with isolate geography. Median indicated by bold dashed line, quartiles denoted by dotted lines.

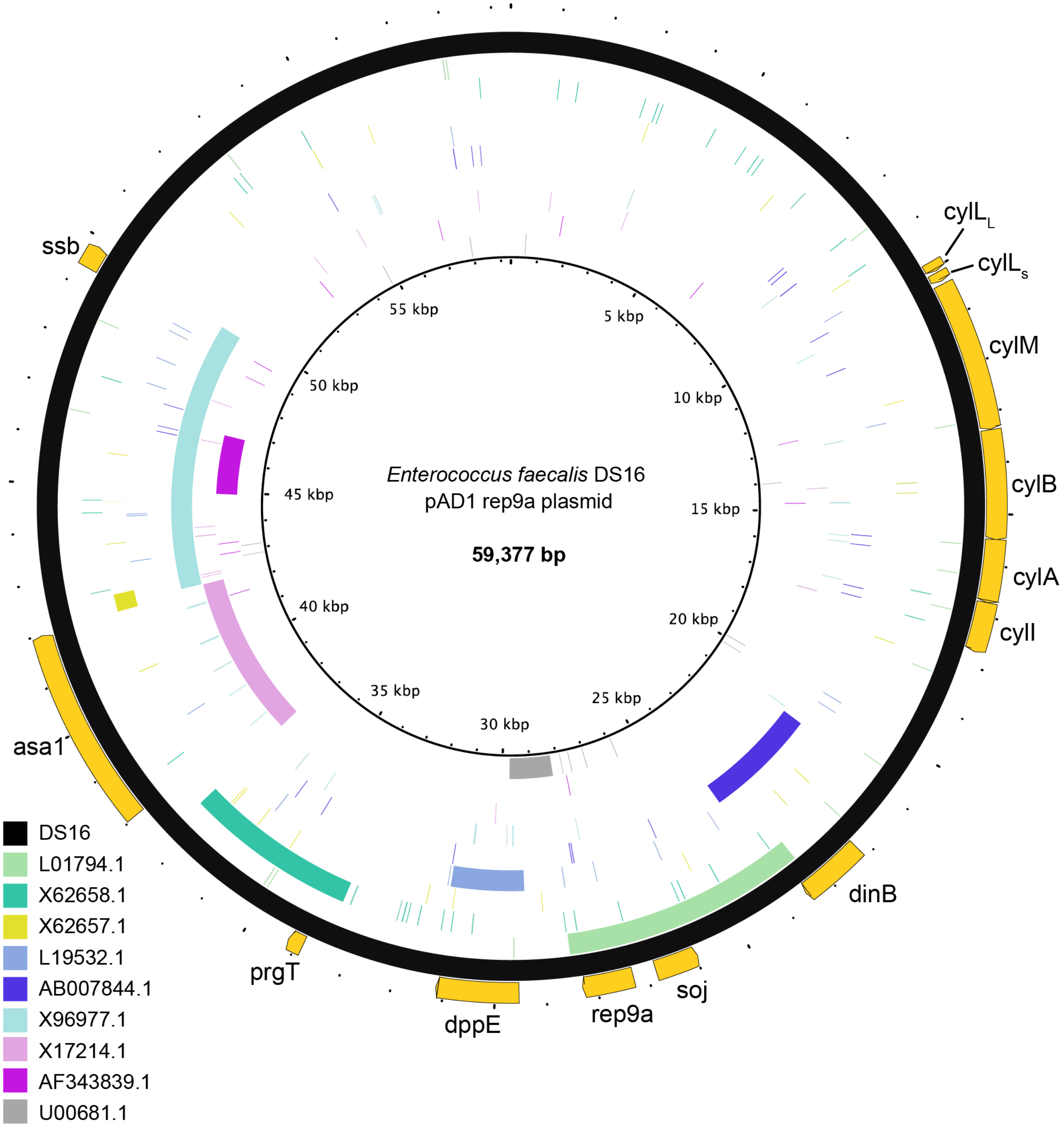

Figure S3. DS16 pAD1 plasmid sequence fragments alignment. Global nucleotide alignment with free end-gaps of publicly available and previously published pAD1 sequence fragments to the newly assembled complete pAD1 plasmid. From outermost to innermost: annotated coding sequences denoted by yellow arrow on outermost ring, black ring represents complete pAD1 sequence, all subsequent rings are aligned fragment sequences to the complete plasmid reference. All displayed alignments possess sequence identity >70%.

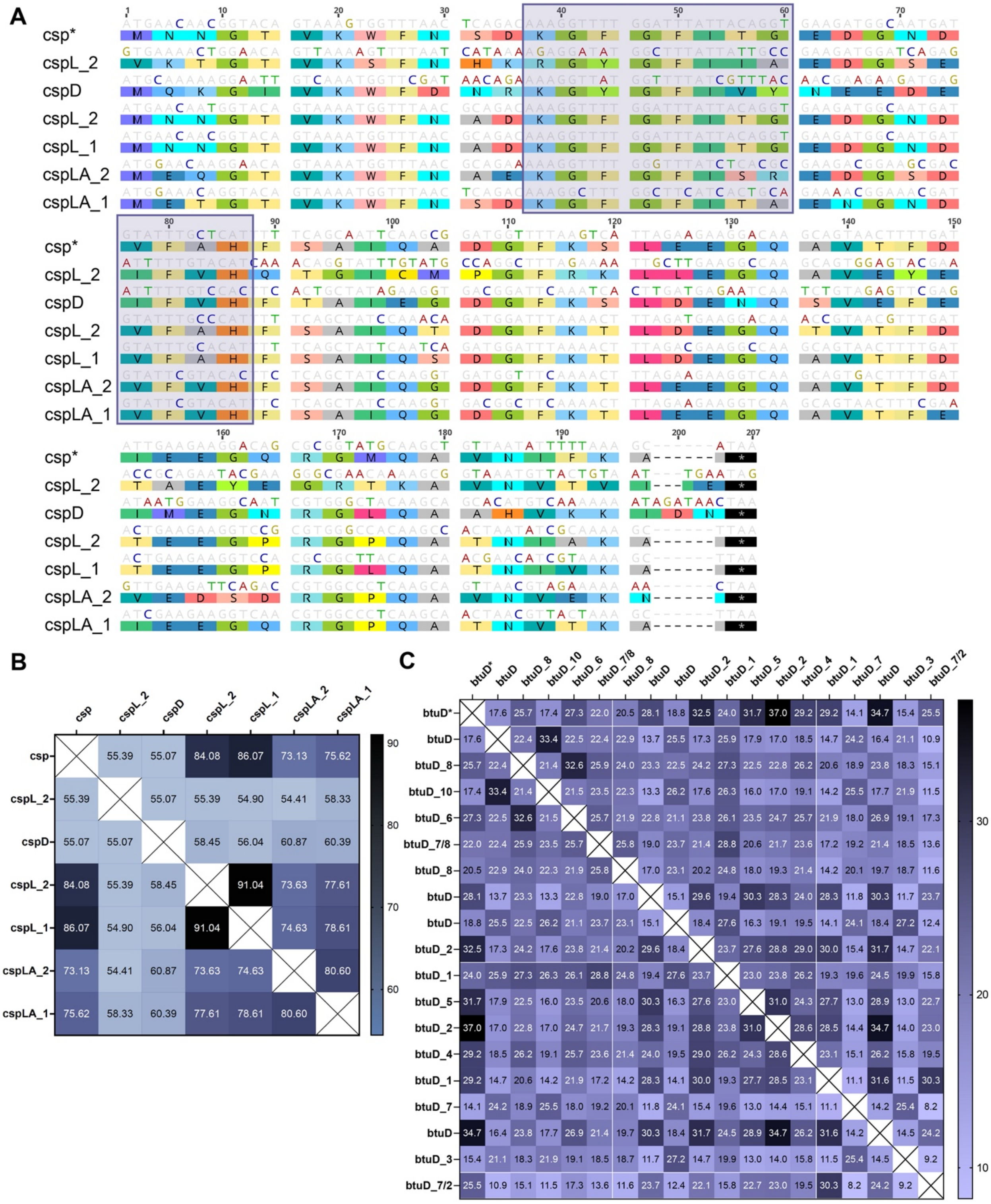

Figure S4. Csp and BtuD paralog alignments. (A) MUSCLE nucleotide alignment of 7 csp alleles. Residues of nucleic acid binding (ribonucleoprotein, RNP, sites) are shaded. **(B)** Sequence identity (%) matrix of all csp alleles. Identity values are listed within heat map. **(C)** MUSCLE nucleotide alignments of representative sequences of 19 btuD alleles identified in the pangenome of E. faecalis suggest the enriched allele is a unique vitamin B12 import ATP binding protein. Identity (%) is annotated. *Enrichment candidate allele.

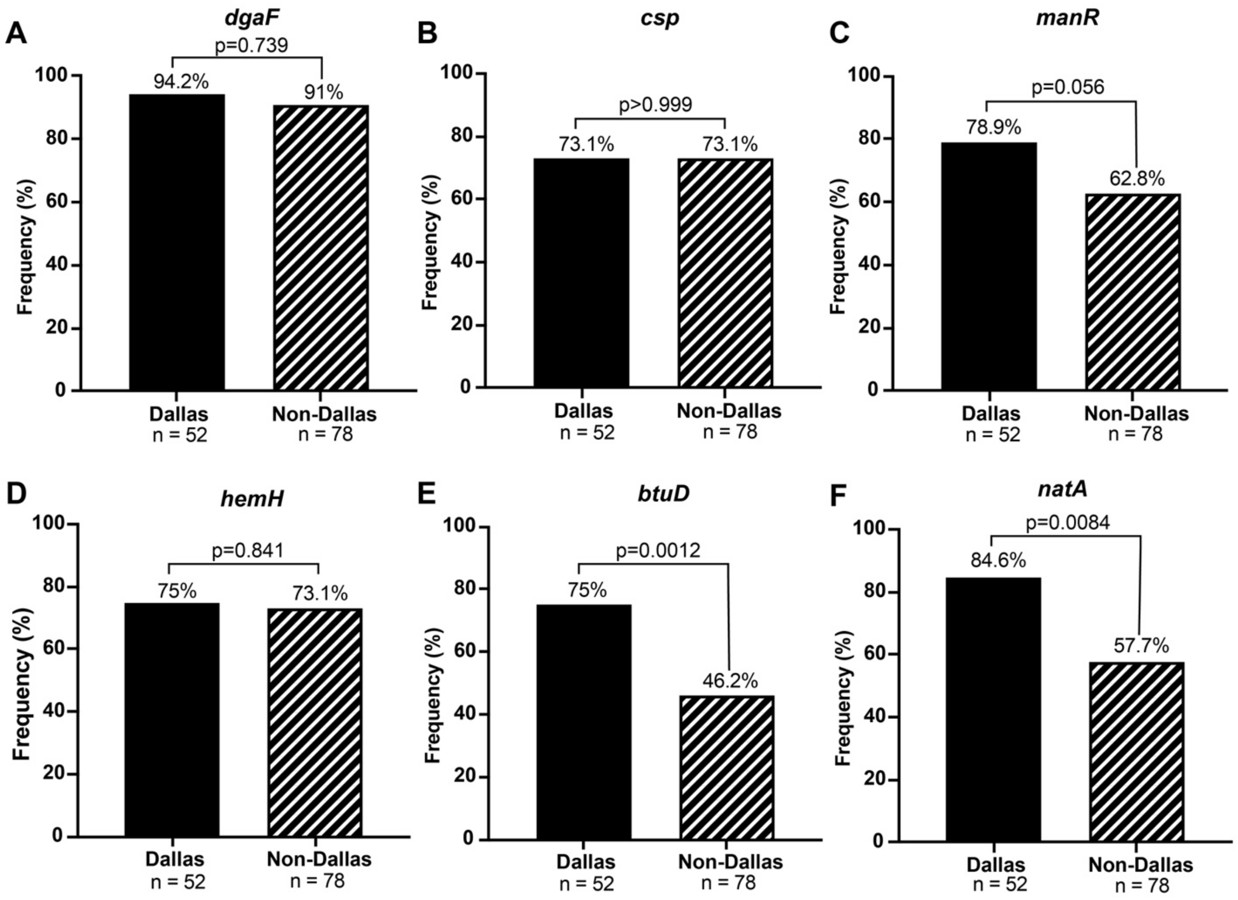

Figure S5. Geographical location association with representative enrichment candidates. (A-F) Frequency of isolates possessing the enrichment candidate within Dallas and Non-Dallas groups. Statistical significance was determined using Fisher's Exact test.

Supplemental References

1. Sharon BM, Hulyalkar NV, Nguyen VH, Zimmern PE, Palmer KL, De Nisco NJ. 2021. Hybrid De Novo Genome Assembly for the Generation of Complete Genomes of Urinary Bacteria using Short- and Long-read Sequencing Technologies. J Vis Exp doi:10.3791/62872.
2. Sharon BM, Nguyen A, Arute AP, Hulyalkar NV, Nguyen VH, Zimmern PE, De Nisco NJ. 2020. Complete Genome Sequences of Seven Uropathogenic Escherichia coli Strains Isolated from Postmenopausal Women with Recurrent Urinary Tract Infection. Microbiol Resour Announc 9.
3. De Coster W, D'Hert S, Schultz DT, Cruts M, Van Broeckhoven C. 2018. NanoPack: visualizing and processing long-read sequencing data. Bioinformatics 34:2666-2669.
4. Wick RR, Judd LM, Gorrie CL, Holt KE. 2017. Unicycler: Resolving bacterial genome assemblies from short and long sequencing reads. PLoS Comput Biol 13:e1005595.
5. Bankevich A, Nurk S, Antipov D, Gurevich AA, Dvorkin M, Kulikov AS, Lesin VM, Nikolenko SI, Pham S, Prjibelski AD, Pyshkin AV, Sirotkin AV, Vyahhi N, Tesler G,

Alekseyev MA, Pevzner PA. 2012. SPAdes: a new genome assembly algorithm and its applications to single-cell sequencing. J Comput Biol 19:455-77.
6. Vaser R, Sovic I, Nagarajan N, Sikic M. 2017. Fast and accurate de novo genome assembly from long uncorrected reads. Genome Res 27:737-746.
7. Walker BJ, Abeel T, Shea T, Priest M, Abouelliel A, Sakthikumar S, Cuomo CA, Zeng Q, Wortman J, Young SK, Earl AM. 2014. Pilon: an integrated tool for comprehensive microbial variant detection and genome assembly improvement. PLoS One 9:e112963.
8. Arkin AP, Cottingham RW, Henry CS, Harris NL, Stevens RL, Maslov S, Dehal P, Ware D, Perez F, Canon S, Sneddon MW, Henderson ML, Riehl WJ, Murphy-Olson D, Chan SY, Kamimura RT, Kumari S, Drake MM, Brettin TS, Glass EM, Chivian D, Gunter D, Weston DJ, Allen BH, Baumohl J, Best AA, Bowen B, Brenner SE, Bun CC, Chandonia JM, Chia JM, Colasanti R, Conrad N, Davis JJ, Davison BH, DeJongh M, Devoid S, Dietrich E, Dubchak I, Edirisinghe JN, Fang G, Faria JP, Frybarger PM, Gerlach W, Gerstein M, Greiner A, Gurtowski J, Haun HL, He F, Jain R, et al. 2018. KBase: The United States Department of Energy Systems Biology Knowledgebase. Nat Biotechnol 36:566-569.
9. Parks DH, Imelfort M, Skennerton CT, Hugenholtz P, Tyson GW. 2015. CheckM: assessing the quality of microbial genomes recovered from isolates, single cells, and metagenomes. Genome Res 25:1043-55.
10. Gurevich A, Saveliev V, Vyahhi N, Tesler G. 2013. QUAST: quality assessment tool for genome assemblies. Bioinformatics 29:1072-5.
11. Antipov D, Hartwick N, Shen M, Raiko M, Lapidus A, Pevzner PA. 2016. plasmidSPAdes: assembling plasmids from whole genome sequencing data. Bioinformatics 32:3380-3387.
12. Francia MV, Haas W, Wirth R, Samberger E, Muscholl-Silberhorn A, Gilmore MS, Ike Y, Weaver KE, An FY, Clewell DB. 2001. Completion of the nucleotide sequence of the Enterococcus faecalis conjugative virulence plasmid pAD1 and identification of a second transfer origin. Plasmid 46:117-27.
13. Galli D, Lottspeich F, Wirth R. 1990. Sequence analysis of Enterococcus faecalis aggregation substance encoded by the sex pheromone plasmid pAD1. Mol Microbiol 4:895-904.
14. Weidlich G, Wirth R, Galli D. 1992. Sex pheromone plasmid pAD1-encoded surface exclusion protein of Enterococcus faecalis. Mol Gen Genet 233:161-8.
15. Hirt H, Wirth R, Muscholl A. 1996. Comparative analysis of 18 sex pheromone plasmids from Enterococcus faecalis: detection of a new insertion element on pPD1 and implications for the evolution of this plasmid family. Mol Gen Genet 252:640-7.
16. Tanimoto K, An FY, Clewell DB. 1993. Characterization of the traC determinant of the Enterococcus faecalis hemolysin-bacteriocin plasmid pAD1: binding of sex pheromone. J Bacteriol 175:5260-4.
17. An FY, Clewell DB. 1994. Characterization of the determinant (traB) encoding sex pheromone shutdown by the hemolysin/bacteriocin plasmid pAD1 in Enterococcus faecalis. Plasmid 31:215-21.
18. Weaver KE, Clewell DB, An F. 1993. Identification, characterization, and nucleotide sequence of a region of Enterococcus faecalis pheromone-responsive plasmid pAD1 capable of autonomous replication. J Bacteriol 175:1900-9.
19. Ozawa Y, Tanimoto K, Fujimoto S, Tomita H, Ike Y. 1997. Cloning and genetic analysis of the UV resistance determinant (uvr) encoded on the Enterococcus faecalis pheromone-responsive conjugative plasmid pAD1. J Bacteriol 179:7468-75.
20. Wayne PCaLSI. 2020. CLSI. Performance Standards for Antimicrobial Susceptibility Testing., 30th ed.

21. Bauer AW, Kirby WM, Sherris JC, Turck M. 1966. Antibiotic susceptibility testing by a standardized single disk method. Am J Clin Pathol 45:493-6.
22. Hudzicki J. 2009. Kirby-Bauer Disk Diffusion Susceptibility Test Protocol. American Society for Microbiology.
23. Tiwari S, Nizet O, Dillon N. 2023. Development of a high-throughput minimum inhibitory concentration (HT-MIC) testing workflow. Frontiers in Microbiology 14.
