# Supplementary material for: Genetic and functional enrichments associated with Enterococcus faecalis isolated from the urinary tract
Source: mBio. 2023 Nov 14;14(6):e02515-23. doi: 10.1128/mbio.02515-23 (PMC10746210; doi:10.1128/mbio.02515-23)
Supplement: Legend — for Data S1. [file mbio.02515-23-s0003.docx]

**Supplemental Data Legend**

**Supplemental Data S1. *E. faecalis* pangenome reference genes.** Representative nucleotide sequences of all genes (core and accessory) in *E. faecalis* pangenome constructed from all 147 strains included in this study. Gene name is listed in header followed by nucleotide sequence and may be used as reference to query *E. faecalis* or other microbial genomes for presence, absence, or variants. Sequences for all enrichment analysis candidates detailed in Figure 6 and Table S7 may be found in this file.
